# Supplementary material for: Robust Preanalytical Performance of Soluble PD-1, PD-L1 and PD-L2 Assessed by Sensitive ELISAs in Blood
Source: Biomedicines. 2022 Oct 11;10(10):2534. doi: 10.3390/biomedicines10102534 (PMC9598997; doi:10.3390/biomedicines10102534)
Supplement: Supplementary file 1 [file biomedicines-10-02534-s001.zip › biomedicines-1841650-supplementary.pdf]

biomedicines

Article

Robust Preanalytical Performance of Soluble PD-1, PD-L1 and PD-L2 Assessed by Sensitive ELISAs in Blood

Kimberly Krueger [1], Zsuzsanna Mayer [1], Marc Kottmaier [2], Miriam Gerckens [3], Stefan Boeck [3], Peter Luppa [4] and Stefan Holdenrieder [1,*]

[1] German Heart Center Munich, Clinics at the Technical University Munich, Institute of Laboratory Medicine, Munich Biomarker Research Center, 80636 Munich, Germany
[2] German Heart Center Munich, Clinics at the Technical University Munich, Department of Cardiovascular Disease, 80636 Munich, Germany
[3] Department of Internal Medicine III, University Hospital Munich-Grosshadern, 81377 Munich, Germany
[4] Department of Clinical Chemistry, University Hospital of the Technical University Munich, 81675 Munich, Germany
* Correspondence: holdenrieder@dhm.mhn.de; Tel.: +0049-89-1218-1010

Abstract: The interaction between programmed death-1 receptor PD-1 and its ligands PD-L1 and PD-L2 is involved in self-tolerance, immune escape of cancer, cardiovascular diseases, and COVID-19. As blood-based protein markers they bear great potential to improve oncoimmunology research and monitoring of anti-cancer immunotherapy. A variety of preanalytical conditions were tested to assure high quality plasma sample measurements: (i) different time intervals and storage temperatures before and after blood centrifugation; (ii) fresh samples and repeated freeze–thaw-cycles; (iii) different conditions of sample preparation before measurement. Concerning short-term stability, acceptable recoveries for PD-1 between 80 and 120% were obtained when samples were kept up to 24 h at 4 and 25 °C before and after blood centrifugation. Similarly, recoveries for PD-L2 were acceptable for 24 h at 4 °C and 6 h at 25 °C before blood centrifugation and up to 24 h at 4 and 25 °C after centrifugation. Variations for PD-L1 were somewhat higher, however, at very low signal levels. Sample concentrations (ng/mL) were neither affected by the freezing process nor by repeated freeze–thaw cycles with coefficients of variation for PD-1: 9.1%, PD-L1 6.8%, and PD-L2 4.8%. All three biomarkers showed good stability regarding preanalytic conditions of sample handling enabling reliable and reproducible quantification in oncoimmunology research and clinical settings of anti-cancer immunotherapy.

Keywords: PD-1, PD-L1, PD-L2, preanalytical validation, ELISA, biomarker

Citation: Krueger, K.; Mayer, Z.; Kottmaier, M.; Gerckens, M.; Boeck, S.; Luppa, P. Robust Preanalytical Performance of Soluble PD-1, PD-L1 and PD-L2 Assessed by Sensitive ELISAs in Blood. *Biomedicines* **2022**, *10*, 2534. https://doi.org/10.3390/biomedicines10102534

Academic Editor: Hiroshi Wakao

Received: 14 July 2022
Accepted: 3 October 2022
Published: 11 October 2022

Publisher's Note: MDPI stays neutral with regard to jurisdictional claims in published maps and institutional affiliations.

Copyright: © 2022 by the authors. Submitted for possible open access publication under the terms and conditions of the Creative Commons Attribution (CC BY) license (https://creativecommons.org/licenses/by/4.0/).

Supplementary Material

Table S1. Centrifugation conditions for the short-term experiment.

Condition	Speed [rcf]	Temperature	Duration [min]
Standard	3000	RT	10
0.5x velocity	1500	RT	10
2x velocity	6000	RT	10

Table S2. Specification of conditions in the sample handling experiment.

Condition	Mixing method	Centrifugation
C1	None	No
C2	Vortexing	No
C3	Pipetting	No
C4	None	Yes
C5	Vortexing	Yes
C6 (=reference)	Pipetting	Yes

Centrifugation conditions: 3000 rcf, room temperature, 2 minutes

Table S3. Coefficients of variation in the matrix comparison experiment for PD-1, PD-L1 and PD-L2.

	PD-1			PD-L1			PD-L2		
**Matrix**	**RT**	**-20°C**	**-80°C**	**RT**	**-20°C**	**-80°C**	**RT**	**-20°C**	**-80°C**
Serum	12	10	4	15	8	13	9	14	12
Heparin	15	19	15	32	14	26	19	16	12
EDTA	6	15	4	16	10	13	10	8	14
Citrate	6	1	10	8	14	22	2	11	10

[%], RT: room temperature

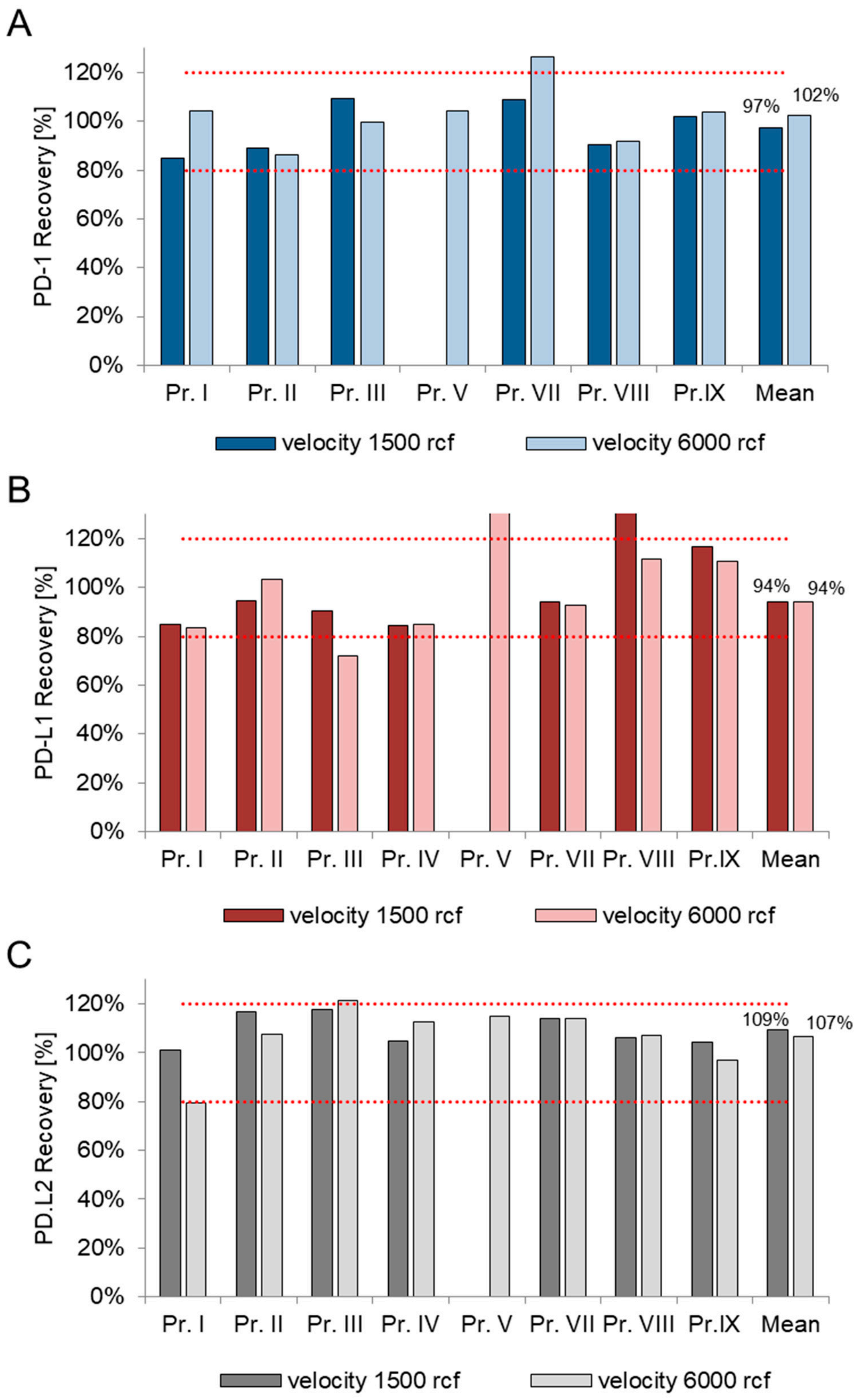

Figure S1. Effect of variation in centrifugation speed on PD-1 (A), PD-L1 (B) and PD-L2 (C) concentrations. Compares the results of different patients at half and double centrifugation speed to the values obtained with centrifugation following laboratory routine (10 min, 3000rcf, RT). Dotted red lines encompass the range between 80% and 120%.

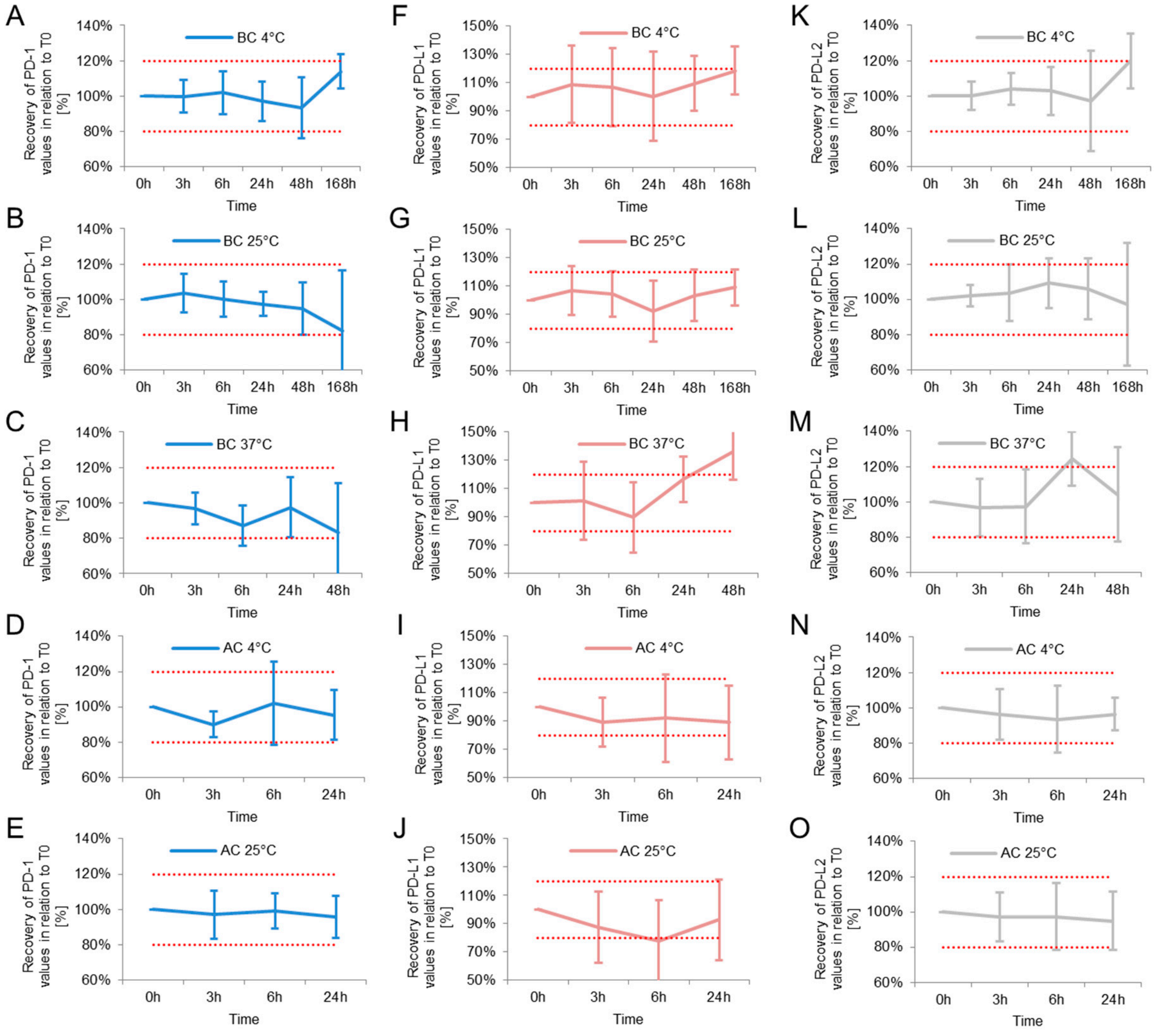

Figure S2. Short-term stability experiment recoveries before and after centrifugation.

The graphs depict the tested five conditions for PD-1 (A-E), PD-L1 (F-J) and PD-L2 (K-O). All graphs summarize the mean value of all included patients. Therefore error bars visualize the corresponding coefficients of variation. Dotted red lines encompass the range between 80% and 120%.

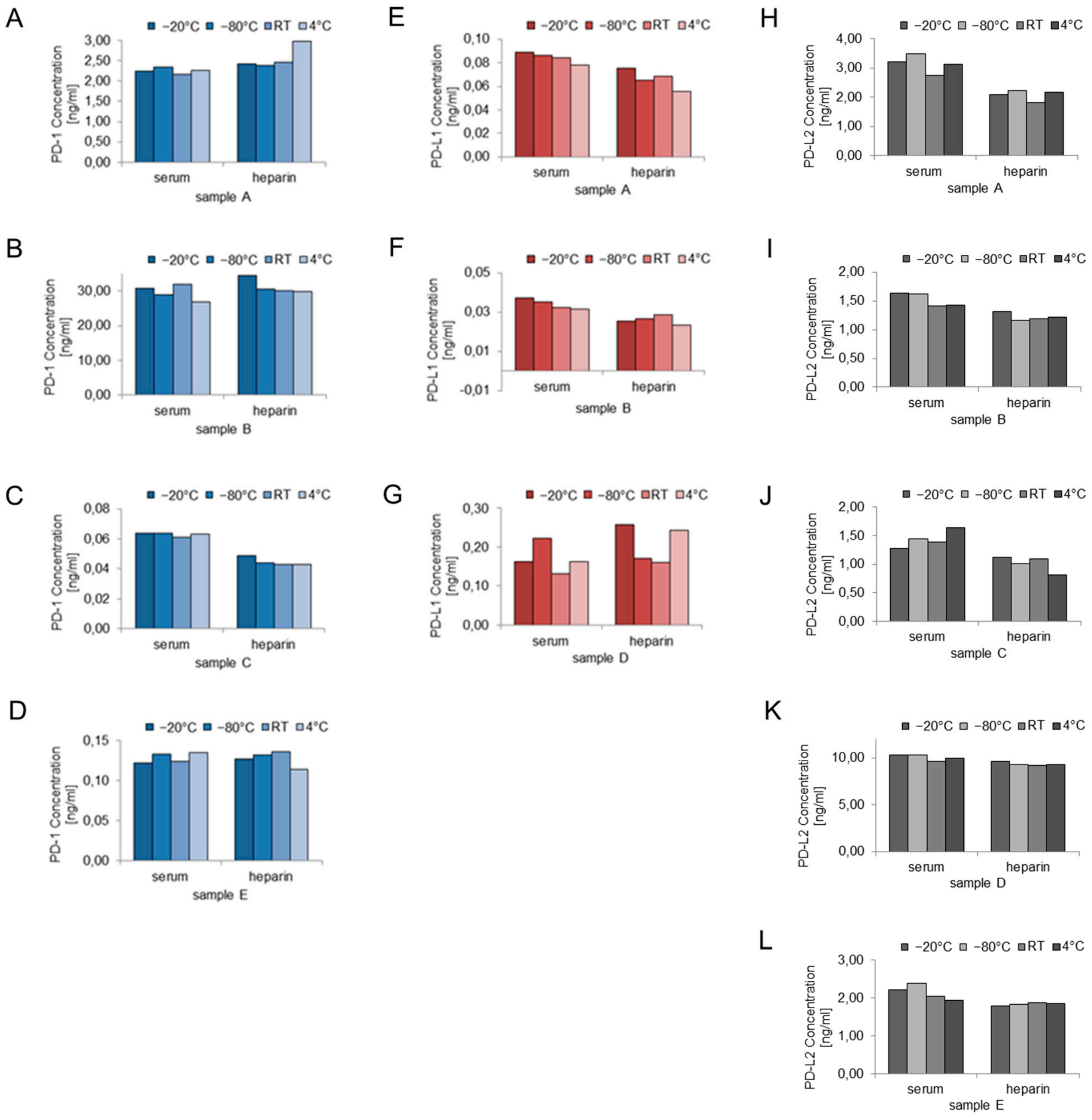

Figure S3. Absolute values of all participants in the freeze experiment.

Absolute concentrations in serum and heparin plasma are presented for PD-1 (A-D), PD-L1 (E-G) and PD-L2 (H-L). RT, room temperature

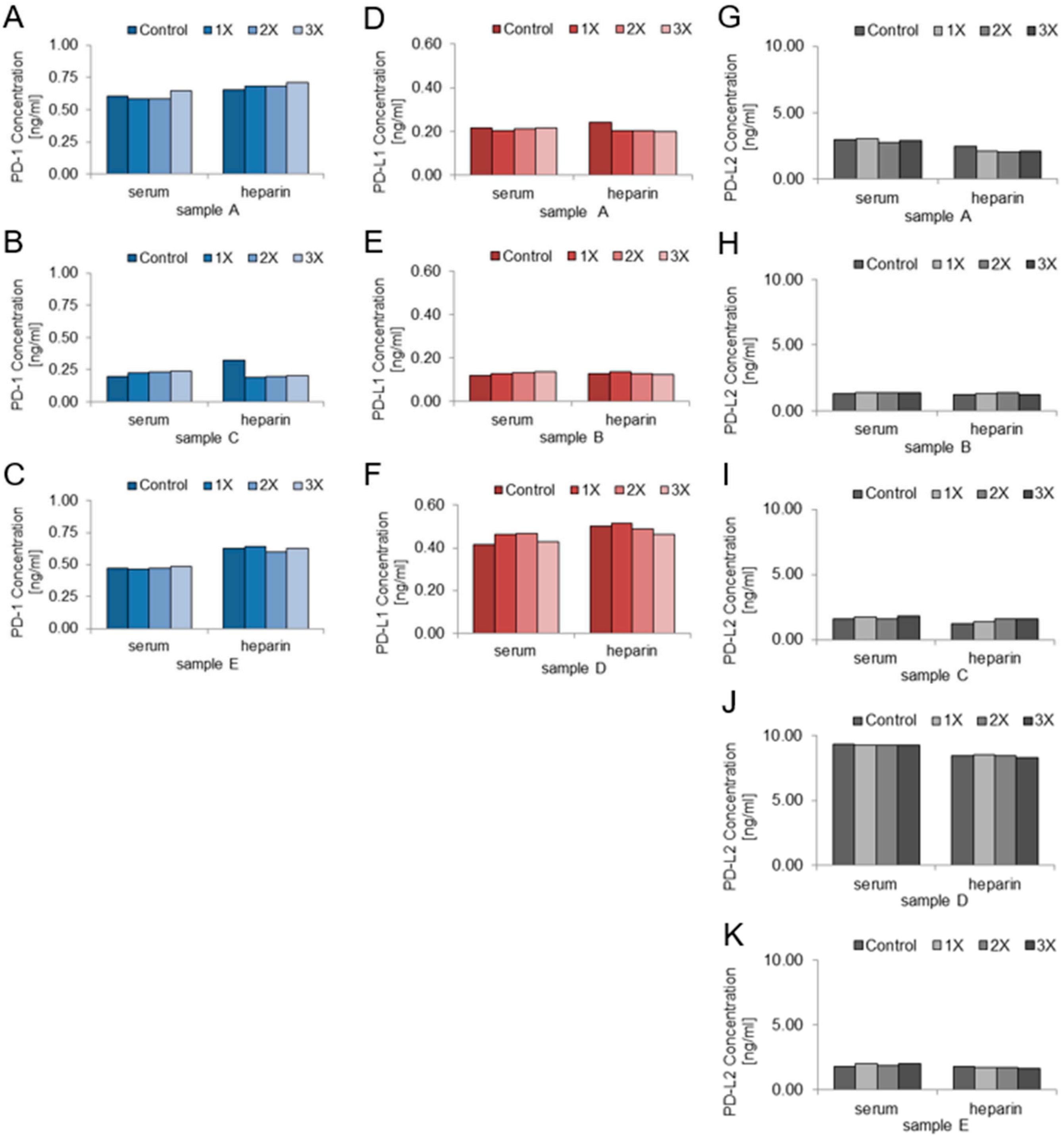

Figure S4. Absolute concentrations of all participants in the freeze-and-thaw experiment. Absolute concentrations in serum and heparin plasma are presented for PD-1 (A-C), PD-L1 (D-F) and PD-L2 (G-K). RT, room temperature

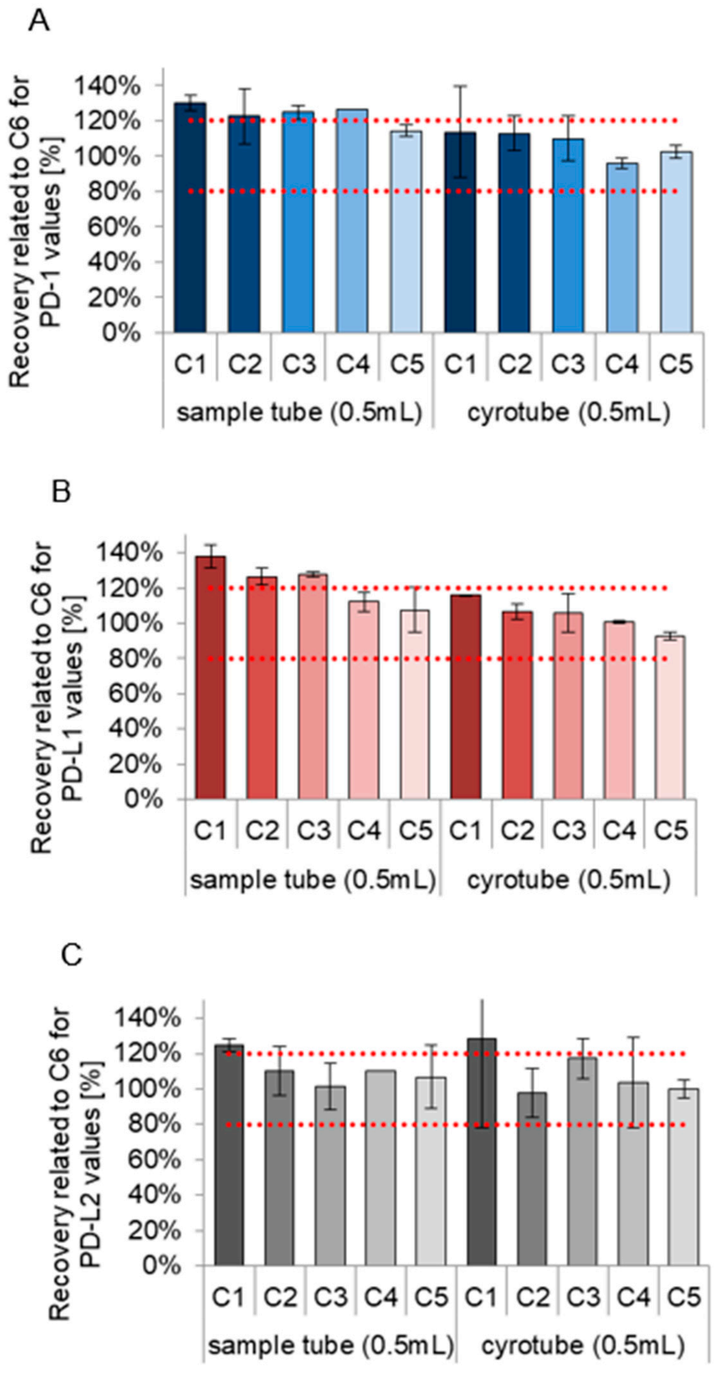

Figure S5. Comparison of two sample storing tubes and different sample preparing conditions for PD-1 (A), PD-L1 (B) and PD_L2 (C). C1: no mixing, no centrifugation, C2: mixing by vortexing, no centrifugation, C3: mixing by pipetting, no centrifugation, C4: no mixing, centrifugation: 2 min, 3000 rcf, RT, C5: mixing by vortexing, centrifugation: 2 min, 3000 rcf, RT, C6: mixing by pipetting, centrifugation: 2 min, 3000 rcf (reference). The figure compares different sample handling approaches before application of the sample on the plate. Samples were stored in two different tubes. This pilot experiment included two patient samples. Dotted red lines encompass the range between 80% and 120%.
